# Silibinin treatment prevents endotoxin-induced uveitis in rats in vivo and in vitro

**DOI:** 10.1371/journal.pone.0174971

**Published:** 2017-04-04

**Authors:** Ching-Long Chen, Jiann-Torng Chen, Chang-Min Liang, Ming-Cheng Tai, Da-Wen Lu, Yi-Hao Chen

**Affiliations:** 1 Graduate Institute of Medical Science, National Defense Medical Center, Taipei, Taiwan; 2 Department of Ophthalmology, Tri-Service General Hospital, National Defense Medical Center, Taipei, Taiwan; National Institutes of Health, UNITED STATES

## Abstract

Uveitis, an intraocular inflammatory disease, occurs mostly in young people and can result in the loss of socioeconomic capabilities. Silibinin has been shown to exert anti-inflammatory effects in human retinal pigment epithelial (RPE) cells. The present study investigated the anti-inflammatory effect of silibinin pretreatment on endotoxin-induced uveitis (EIU) in rats and the mechanisms by which it exerts these effects. Uveitis was induced via injection of lipopolysaccharides (LPS) into Lewis rats. Twenty-four hours after the LPS injection, histological examination showed that silibinin decreased inflammatory cell infiltration in the anterior segment of the eyes of LPS-treated rats. Analyses of the aqueous humor showed that silibinin decreased cell infiltration, protein concentration, nitric oxide (NO), and prostaglandin (PG)-E2 production. Western blot analysis indicated that silibinin decreased the expression of inducible NO synthase (iNOS), cyclooxygenase (COX-2), and phosphorylated IkB in the iris-ciliary body (ICB). Immunohistochemistry showed that silibinin decreased intercellular adhesion molecule (ICAM-1) expression in the ICB. In addition, western blot analysis showed that silibinin attenuated the expression of iNOS, COX-2, ICAM-1, and nuclear p65 in LPS-treated RAW cells. In conclusion, silibinin pretreatment prevents EIU and the subsequent production of proinflammatory mediators and ICAM-1, at least in part, by blocking the NF-κB–dependent signaling pathway both *in vivo* and *in vitro*. These effects may contribute to the silibinin-mediated preventive effects on intraocular inflammatory diseases such as acute uveitis.

## Introduction

Uveitis is an intraocular inflammatory disease that can affect any part of the eye and cause serious complications. It accounts for 10–15% of the number of cases of total blindness and up to 20% of the cases of legal blindness in developed countries.[1, 2] It occurs mostly in young people and can result in loss of the patient's independence and socioeconomic capabilities.[3] Currently, the therapeutic strategy to combat uveitis is to suppress inflammation and therefore corticosteroids are the mainstay of therapy;[4] however, corticosteroids can cause many unwanted ocular side effects, including accelerated cataract formation, increased intraocular pressure,[5, 6] and systemic side effects such as hypertension, diabetes, Cushing’s syndrome, and osteoporosis.[7] Therefore, investigating the mechanisms of intraocular inflammation and developing effective preventive agents for uveitis remain important issues.

Endotoxin-induced uveitis (EIU) in animals is an animal-based model to study uveitis and is an acute form of uveitis that can be induced in rats, mice, and rabbits by exposure to a sub-lethal dose of exogenous bacterial toxins, such as lipopolysaccharides (LPS).[8–10] Systemic injection of LPS can generate inflammatory responses, largely in the anterior uvea, and mild responses in the posterior segments of the eye, both of which mimic the pathological conditions of human acute uveitis.[8, 11, 12] EIU is a widely used experimental model to investigate the pathological mechanism of ocular inflammation and test the efficacy of potential anti-inflammatory agents.[13] The clinically relevant classical signs of inflammation in EIU are intense acute, but transient, cellular infiltration in the anterior and vitreous chambers by neutrophils and macrophages,[8, 14] in which acute inflammatory responses occur 4 h after the LPS injection, reach a peak at 18 to 24 h, and are maintained for 72 h.[14, 15] Furthermore, recent studies have shown that inflammatory mediators such as nitric oxide (NO)[16, 17] and prostaglandin (PG)-E2[17], and, cell-adhesion molecules such as intercellular adhesion molecule (ICAM)-1[14, 18] are involved in the pathogenesis of EIU. The activation of transcription factor nuclear factor-κB (NF-κB) plays a key role in LPS-dependent inducible gene expression, which results in the production and release of inflammatory mediators and adhesion molecules.[19, 20] Therefore, the suppression of NF-κB activation could be a potential therapeutic target for ocular inflammation.

Silibinin, a hepatoprotective medicine, is the main active component of the silymarin complex extracted from milk thistle. Silibinin has antioxidant and tissue regenerative properties that have wide applications in hepato-, neuro-, nephro-, and cardio-protection.[21] Recently, researchers have focused on the anti-cancer effects of silibinin through multiple molecular mechanisms, and, its use as a preventive and therapeutic agent in cancer therapy has been approved based on its potential usefulness.[22] In addition, Ramasamy et al. demonstrated that the anti-inflammatory effects of silymarin are related to the inhibition of the transcription factor NF-κB that regulates inflammation- and immune response-related gene expression.[22] In our previous study, we also demonstrated that silibinin inhibits ICAM-1 expression, and thereby its function, by suppressing the NF-κB signaling pathway in TNF-α and IFN-γ -stimulated retinal pigment epithelial (RPE) cells.[23] Therefore, these results prompted us to investigate the effect of silibinin pretreatment on EIU. The objective of this study was to investigate the potential use of silibinin in preventive therapy against EIU *in vivo* and *in vitro*.

## Materials and methods

### Animals and induction of uveitis

All animal experiments were approved and conducted under the guidance of the Institutional Animal Care and Use Committee (accredited by the Association for Assessment and Accreditation of Laboratory Animal Care International) of the National Defense Medical Center, Taipei, Taiwan (No: IACUC-13-135). All animals used in the study were cared for in accordance with the Association for Research in Vision and Ophthalmology Statement for the Use of Animals in Ophthalmic and Vision Research. Eight-week-old male Lewis rats (210–220 g) were used in this study (purchased from LASCO Co., Charles River Technology, Taipei, Taiwan). They were maintained under a 12-hour light/12-hour dark cycle. Food and water were supplied ad libitum.

To induce uveitis, LPS from *Salmonella typhimurium* (Sigma-Aldrich, St. Louis, MO, USA) diluted in 0.2 ml of phosphate-buffered saline (PBS; pH 7.4) was injected into the footpad of each rat at a dose of 300 μg/kg body weight (BW). Silibinin (Sigma-Aldrich, St. Louis, MO, USA) that had been dissolved in 0.2 ml of dimethyl sulfoxide (DMSO) was administered by intraperitoneal injection (i.p.) to each rat at a dose of 100 and 200 mg/kg BW.

Rats were randomly allocated to the following five groups: (1) A control group, consisting of rats that received i.p. injections of 0.2 ml DMSO daily for 3 days, and then the footpad was injected with 0.2 ml PBS for 24 h; (2) A silibinin group, consisting of rats that received daily i.p. injections of Silibinin (200 mg/kg in 0.2 ml DMSO) for 3 days, and then the footpad was injected with 0.2 ml PBS for 24 h; (3) an LPS group, consisting of rats that received daily i.p. injections of 0.2 ml DMSO for 3 days, and then the footpad was injected with LPS (300 μg/kg in 0.2ml PBS) for 24 h; (4) a silibinin (100)+LPS group, consisting of rats that received daily i.p. injections of silibinin (100 mg/kg in 0.2 ml DMSO) for 3 days, and then the footpad was injected with LPS (300 μg/kg in 0.2 ml PBS) for 24 h; (5) a Silibinin (200)+LPS group, consisting of rats that received daily i.p. injections of silibinin (200 mg/kg in 0.2 ml DMSO) for 3 days, and then the footpad was injected with LPS (300 μg/kg in 0.2 ml PBS) for 24 h. There were 16 rats in each group, of which 4 were used for the collection of aqueous humor (one eye was used for cell counting and the other for the protein concentration assay), another 4 were used for the collection of additional aqueous humor (one eye was used for measuring of NO levels and the other for measuring of PG-E2 levels), 4 were used for the preparation of iris-ciliary body (ICB) lysates, and the remaining 4 were used for histological and immunohistological analyses. 24 h after the LPS injection, euthanasia of rats was conducted in CO_2_ chambers.

### Cell counts and protein concentration

Cell counting and measurement of protein concentration in aqueous humor (AqH) were performed as previously described.[14] Briefly, immediately after euthanization, the AqH was collected by puncturing the anterior chamber of the eye with a 30-gauge needle. For cell counting, the AqH was mixed with an equal amount of trypan blue solution (Sigma-Aldrich, St. Louis, MO, USA), and 1 drop of the cell suspension was applied to a hemocytometer. The number of cells per square (equivalent to 0.1 μL) was counted manually using a light microscope, and the mean number of cells counted from five squares per sample was multiplied by two to correct the previous dilution. A BCA (bicinchoninic acid) protein assay reagent kit (Pierce, Rockford, IL, USA) was used to measure total protein concentration in the AqH.

### Histology and immunohistology

For histology and immunohistology, eyes were enucleated immediately after death and immersed in 4% paraformaldehyde for at least 24 h, after which they were snap-frozen in liquid nitrogen and embedded in OCT compound in cryomolds. More than 20 serial axial cryostat sections (6 μm thick) were cut from each eye, starting at the optic nerve head.

Sections were stained with hematoxylin and eosin for histological studies. For immunohistology, sections were deparaffinized in xylene prior to rehydration using gradient alcohol, and endogenous peroxidase activity was blocked by treatment with 3% hydrogen peroxide for 20 min. Antigen retrieval was performed by treating the sections with citrate buffer saline (pH 6.0) for 15 min at 95°C in a microwave oven. Non-specific binding was prevented by blocking with 10% bovine serum albumin (BSA) for 30 min at room temperature. Sections were incubated with primary antibodies for ICAM-1 (diluted 1:200 in TBST; Santa Cruz Biotechnology, Santa Cruz, CA, USA) for another 60 min at room temperature. Following incubation, sections were lavaged with tris-buffered saline (TBS) and incubated with goat anti-rabbit IgG conjugated with horseradish peroxidase for 30 min at room temperature. Staining was performed using 3,3'-diaminobenzidine (DAB) as the chromogen, and sections were counterstained with hematoxylin followed by dehydration and mounting. Negative controls were prepared using TBS in lieu of the first antibody. The stained slides were observed and analyzed under a microscope at 200X magnification.

### Determination of NO and PG-E2 levels in the AqH

The concentrations of NO and PG-E2 in the AqH were detected by an NO quantitation kit (Active Motif, Carlsbad, CA, USA) and a PG-E2 ELISA kit (Neogen, Lansing, MI, USA), respectively, following the manufacturer's instructions. Four independent replications of each experiment were performed.

### Preparation of rat ICB lysates

Rat ICB lysates were prepared in the following manner. Briefly, in each group, the ICBs were carefully isolated and homogenized in 50 μL of hypotonic buffer (10 mM HEPES-KCl, 1 mM β-mercaptoethanol, and 1 mM dithiothreitol). After incubation on ice for 10 min, the homogenate was vortexed for 10 s and centrifuged at 1000 g. The supernatant was discarded, the pellet was resuspended in 100 μL of lysis buffer in the presence of protease inhibitors and was incubated on ice for 10 min. The insoluble debris of rat ICB lysates was removed by centrifugation at 12,000 g at 4°C for 15 minutes.

### Cell culture and treatment

Mouse macrophage-like RAW 264.7 cells were obtained from the Bioresource Collection and Research Center (Taipei, Taiwan) and were cultured in Dulbecco’s modified Eagle’s medium (DMEM) supplemented with 4 mM _L_-glutamine, 10% FBS (fetal bovine serum), 100 U/mL of penicillin, and 100 mg/mL of streptomycin at 37°C in a humidified atmosphere containing 5% CO_2_. The confluent RAW cells were pretreated with 50 and 100 μM silibinin for 18 h and then co-treated for 24 h with 100 ng/mL of LPS derived from *S*. *typhimurium* (Sigma-Aldrich, St. Louis, MO, USA), unless otherwise stated.

### Preparation of RAW cell lysates

RAW cell lysates were prepared as follows. Briefly, confluent cultured cells were pretreated with or without 50 and 100 μM of silibinin, and co-treated with LPS for 24 h at 37°C. The cells were pelleted at 1000 g, resuspended, and sonicated in cold lysis buffer (50 mM Tris-HCl [pH 7.5], 2% sodium dodecyl sulfate (SDS), and 1 mM phenylmethylsulfonyl fluoride. The insoluble debris of RAW cell lysates was removed by centrifugation at 12,000 g at 4°C for 15 min.

### Preparation of nuclear extract from RAW cells

Nuclear extraction of proteins from RAW cells was performed as follows. Confluent cultured cells were pretreated with or without silibinin (50 and 100 μM) and co-treated with LPS for 24 h at 37°C. The cells were washed twice with PBS. Proteins were extracted from the nucleus by using a Nuclear Extraction Kit (Affymetrix, Santa Clara, CA, USA) and following the manufacturer's instructions.

### Western blot analysis

The protein content of rat ICB lysates, RAW cell lysates, and nuclear extracts was determined using the BCA method (BCA; Pierce, Rockford, IL, USA) with BSA (bovine serum albumin) as the standard. The lysates (20 *μ*g) were resolved using one-dimensional SDS–polyacrylamide gel electrophoresis (SDS-PAGE). The separated proteins were transferred onto polyvinylidene difluoride (PVDF) membranes (Immobilon; Millipore, Bedford, MA, USA), blocked with 5% (w/v) skim milk for 1 h at room temperature, and then incubated overnight at 4°C with antibodies directed against iNOS (diluted 1:1,000 in Tris-buffered saline containing Tween-20 (TBST, 0.1% at 1X; Santa Cruz Biotechnology, Santa Cruz, CA, USA), COX-2 (diluted 1:1,000 in TBST; Santa Cruz Biotechnology), rat ICAM-1 (diluted 1:1,000 in TBST; R&D Systems, Minneapolis, MN, USA), IkB (diluted 1:1,000 in TBST; Santa Cruz Biotechnology), p-IkB (diluted 1:1,000 in TBST; Cell Signaling Technology, Danvers, MA, USA), p65 (diluted 1:1,000 in TBST; Santa Cruz Biotechnology), glyceraldehyde 3-phosphate dehydrogenase (GAPDH; diluted 1:5,000 in TBST; Santa Cruz Biotechnology), α-tubulin (diluted 1:5,000 in TBST; Santa Cruz Biotechnology), and Lamin B1 (diluted 1:1,000 in TBST; Abcam, Cambridge, UK). The membranes were washed and incubated with horseradish-peroxidase-conjugated secondary antibodies (1:25,000; Jackson ImmunoResearch Laboratories, Pennsylvania, USA) for 1 h at room temperature, and the protein was visualized using an enhanced chemiluminescence (ECL) procedure (enhanced chemiluminescence reagent; Millipore, Billerica, MA, USA). The images of western blotting were acquired with a UVP BioSpectrum 500 and analyzed by Vision Works LS software (UVP, California, USA).

### Statistical analysis

Data were analyzed using a one-way analysis of variance (ANOVA). When a significant difference between the groups was obtained, multiple comparisons of their means were made with Tukey’s post hoc test to identify which group was significantly different. Data are presented as means ± standard deviations (SDs). Each result is representative of at least three independent experiments. Differences in means were deemed to be significant when P ≤ 0.05. Statistical analyses were performed using the GraphPad Prism version 5.01 for Windows (GraphPad Software, La Jolla, California, USA).

## Results

### Effects of silibinin on the histopathological changes in the anterior segment of the eyes of rats with EIU

**Fig 1:**
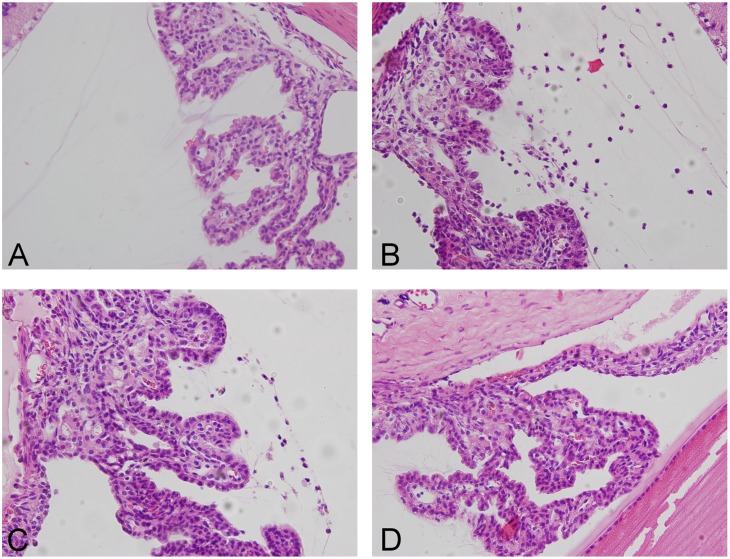
Histopathological features of inflammatory cell infiltration in the anterior segment of rat eyes. (A) Control group showed no cellular infiltration in the histopathological section of the anterior segment. (B) LPS group showed severe inflammatory cell infiltration compared to the control group. (C) Silibinin (100)+ LPS group showed a decreased amount of inflammatory cells compared to the LPS group. (D) Silibinin (200)+ LPS group showed a significantly decreased amount of inflammatory cells compared to the LPS group. These images are representative of four rats in each group. Cells were stained with hematoxylin and eosin and viewed under 200X magnification.

To investigate the effect of silibinin pretreatment on ocular inflammation in LPS-treated rats *in vivo*, the histopathological changes in the anterior segment of eyes were examined after staining with hematoxylin and eosin. As shown in Fig 1, the results indicated that no cellular infiltration was observed in the anterior segment of the control group (Fig 1A). Conversely, in the LPS group, histological evaluation revealed massive cell infiltration, predominantly into the anterior segment as compared to the control group (Fig 1B). Compared with the LPS group, the silibinin (100)+ LPS group showed a reduction of cellular infiltration in the anterior segment (Fig 1C); however, there was significantly reduced cellular infiltration in the silibinin (200)+ LPS group (Fig 1D). Therefore, these results show that silibinin attenuates LPS-induced histopathological changes in the anterior segment in a dose-responsive manner.

### Effects of silibinin on LPS-induced cellular infiltration and protein concentration in the AqH

**Fig 2:**
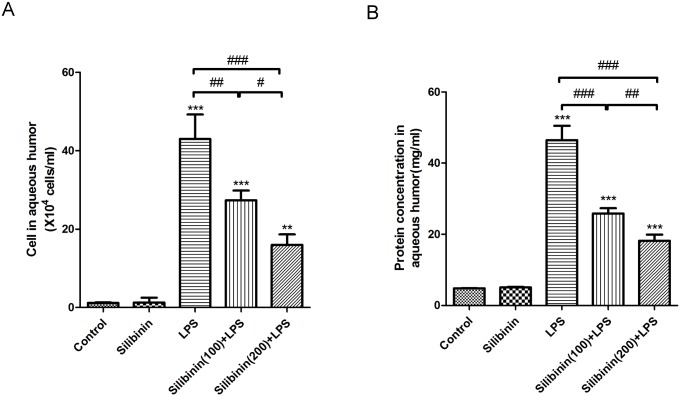
Effects of silibinin on Lipopolysaccharide (LPS)-induced cellular infiltration (A) and protein concentration (B) in the aqueous humor of rats. In each group, the aqueous humor was collected to count cells and measure protein concentration. Compared to the control group, the Silibinin group did not affect the rates of cellular infiltration or protein concentrations. In the LPS group, the cell counts and protein concentration markedly increased; however, the Silibinin (100)+ LPS group and Silibinin (200)+ LPS group significantly reduced these effects in a dose-responsive manner. Each value represents the mean ± SD of data of four rats. ***P < 0.001 versus the control group; ^#^*P* < 0.05; ^##^*P* < 0.01; ^###^*P* < 0.001.

As described above, the indicated dosages of silibinin attenuated the LPS-induced histopathological changes in the anterior segment of the eye (Fig 1). To investigate the effect of silibinin pretreatment on the cellular infiltration and protein concentration in the AqH in LPS-treated rats, we collected the AqH to count cells and measure protein concentration. As shown in Fig 2A, in the LPS group, the number of the infiltrated cells in the AqH showed a significant increase (43 ± 6.25 x10^4^/mL; P < 0.001) compared to the control group. In the Silibinin (100)+ LPS group and Silibinin (200)+ LPS group, the infiltrated cells in the AqH significantly decreased (P < 0.01 and P < 0.001, respectively) in a dose-responsive manner (27.33 ± 2.52 x10^4^/mL and 16 ± 2.65 x10^4^/mL, respectively), compared to those in the LPS group. Similarly, protein concentration in the AqH of the LPS group increased significantly (46.47 ± 4.03 mg/mL; P < 0.001) compared to the control group; however, the Silibinin (100)+ LPS group and Silibinin (200)+ LPS group significantly attenuated this increase (P < 0.001 for each) in the protein concentration in the AqH in a dose-responsive manner (25.83 ± 1.51 mg/mL and 18.17 ± 1.76 mg/mL respectively; Fig 2B), when compared to the LPS group. These results suggest that silibinin attenuates LPS-induced cellular infiltration and increased protein concentration in the AqH.

### Effects of silibinin on the levels of NO and PG-E2 in the AqH of LPS-treated rats

**Fig 3:**
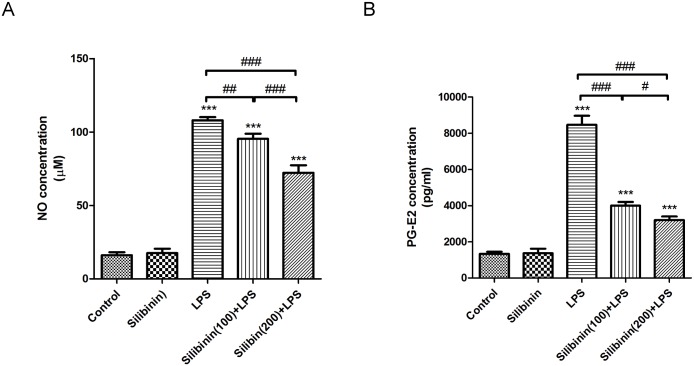
Effects of silibinin on Lipopolysaccharide (LPS)-induced Nitric Oxide (NO) (A) and Prostaglandin (PG)-E2 (B) expression in the aqueous humor of rats. In each group, the aqueous humor was collected to quantify the levels of NO and PG-E2 using a commercially available NO quantitation kit and an ELISA kit respectively. Compared to the control group, the levels of NO and PG-E2 expression increased significantly in the LPS group. Compared to the LPS group, the Silibinin (100)+LPS group and Silibinin (200)+LPS group had significantly decreased expression of LPS-induced NO and PG-E2 in a dose-responsive manner. Each value represents the mean ± SD of results in four rats. ***P < 0.001 versus the control group; ^#^*P* < 0.05; ^##^*P* < 0.01; ^###^*P* < 0.001.

Previous studies have reported that the inflammatory mediators NO and PG-E2 play an important role in LPS-induced intraocular inflammation in EIU.[17] Therefore, we investigated the effects of silibinin pretreatment on the levels of NO and PG-E2 in the AqH of LPS-treated rats. As shown in Fig 3A, in the LPS group, the levels of NO in the AqH of rats significantly increased (108.05 ± 2.08 μM; P < 0.001) compared to the control group. Compared to the LPS group, rats in the Silibinin (100)+ LPS group and Silibinin (200)+ LPS group showed a significant decrease in the levels of NO in the AqH in a dose-responsive manner (95.35 ± 3.51 and 72.25 ± 5.13μM; P < 0.01 and P < 0.001, respectively). Similarly, the levels of PG-E2 were significantly increased (8466.67 ± 503.32 pg/mL; P < 0.001) in the LPS group when compared with those in the control group. Compared to the LPS group, rats in the Silibinin (100)+ LPS group and Silibinin (200)+ LPS group had a significant decrease in the levels of PG-E2 in the AqH (4000 ± 200 and 3200 ± 200pg/mL, respectively; P < 0.001 for each; Fig 3B). Overall, these results demonstrate that silibinin reduces the production of the LPS-induced inflammatory mediators NO and PG-E2 in the AqH.

### Effects of silibinin on LPS-induced expression of iNOS and COX-2 in the ICBs

**Fig 4:**
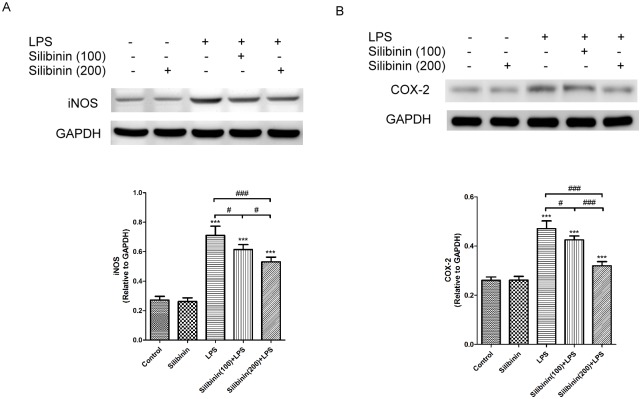
Effects of silibinin on Lipopolysaccharide (LPS)-induced Nitric Oxide Synthase (iNOS) (A) and Cyclooxygenase (COX)-2 (B) protein expression in the Iris-Ciliary Body (ICB) of rats. For each group, the rat ICBs were collected and rat ICB lysates were prepared and analyzed by immunoblotting/western blotting using antibodies against iNOS, COX-2, and glyceraldehyde 3-phosphate dehydrogenase (GAPDH; to normalize protein expression). The optical density of the protein bands for iNOS, COX-2, and GAPDH were analyzed. The data are presented as mean ± SD of four rats per group. The differences in the iNOS and COX-2 protein levels in ICBs of rats from the groups were compared using an ANOVA, followed by Tukey’s post hoc test. ** *P* < 0.01 and *** *P* < 0.001 versus the control group; ^#^ *P* < 0.05; ^###^ *P* < 0.001.

iNOS and COX-2 are primarily responsible for increased NO and PG-E2 production during inflammation.[24, 25] Considering that silibinin reduced the production of LPS-induced NO and PG-E2 in the AqH (Fig 3), it could decrease the expression of iNOS and COX-2 in the ICBs of LPS-treated rats. We used western blot analysis to determine the effects of silibinin pretreatment on the expression of LPS-induced iNOS and COX-2 in the ICB. As shown in Fig 4, we found that the expression of iNOS and COX-2 increased in the ICBs of the LPS group when compared with their expression in the control group. Compared to the LPS group, rats in the Silibinin (100)+ LPS group and Silibinin (200)+ LPS group showed a marked decrease in iNOS and COX-2 protein expression in a dose-responsive manner. Therefore, these results suggest that silibinin decreases the expression of LPS-induced iNOS and COX-2 in the ICB of rats.

### Effect of silibinin on ICAM-1 expression in the ICBs of LPS-treated rats

**Fig 5:**
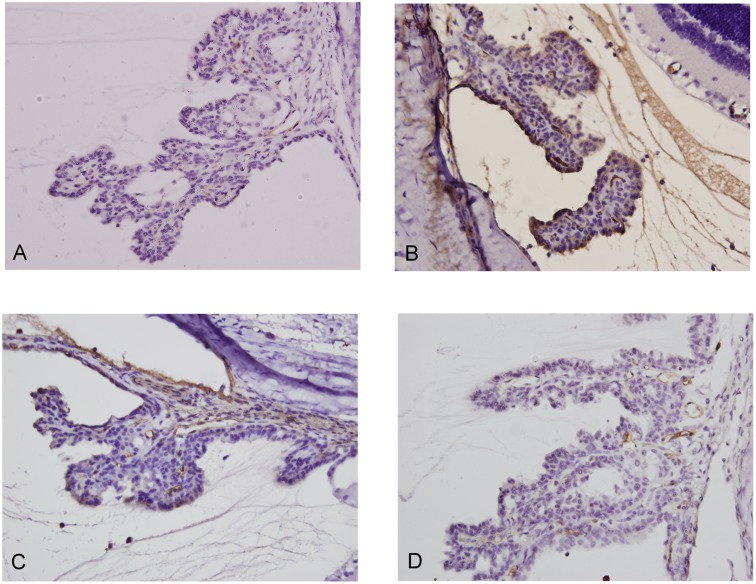
Effects of silibinin on Lipopolysaccharide (LPS)-induced Intercellular Adhesion Molecule (ICAM)-1 expression in the ICBs of rats by Immunohistochemical (IHC) staining. Histopathological sections stained with antibodies against ICAM-1. (A) Control groups. (B) LPS group. (C) Silibinin (100)+ LPS group. (D) Silibinin (200)+ LPS group. The increased expression of ICAM-1 was noted in the LPS group (B) relative to the expression in control group (A). Compared to the LPS group (B), the decreased expression of ICAM-1 was noted in the Silibinin (100)+ LPS group and Silibinin (200)+ LPS group (C, D). These images are representative of four rats in each group. Original magnification 200X.

Our previous studies have reported that ICAM-1 expression plays a critical role in cell infiltration in EIU.[14, 26] In the aforementioned studies, we used immunohistochemical (IHC) staining to evaluate the effects of silibinin pretreatment on ICAM-1 expression in the ICBs of LPS-treated rats. As shown in Fig 5, we found that the ICBs expressed low levels of ICAM-1 in the control group (Fig 5A); however, in the LPS group, the ICBs expressed high levels of ICAM-1 compared to control group (Fig 5B). Compared to the LPS group, the expression of ICAM-1 was slightly decreased in the Silibinin (100)+ LPS group (Fig 5C) and markedly decreased in the Silibinin (200)+ LPS group (Fig 5D). These results indicate that silibinin attenuates LPS-induced ICAM-1 expression in the ICB of rats in a dose-responsive manner.

### Effect of silibinin on the activation of NF-κB in the ICBs of LPS-treated rats

**Fig 6:**
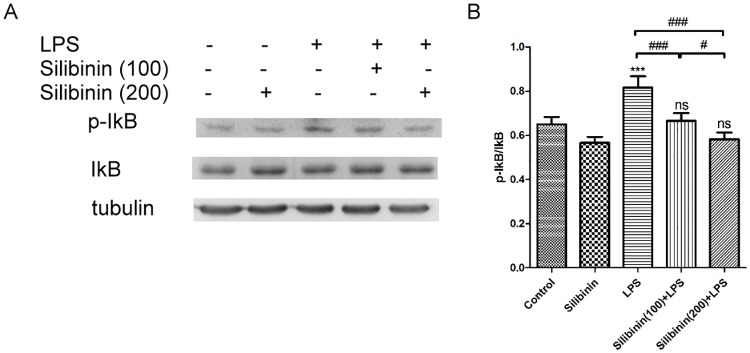
Effects of silibinin on the expression of phosphorylated IkB in the Iris-Ciliary Body (ICB) of Lipopolysaccharide (LPS)-treated rats. (A) For each group, rat ICBs were collected, and rat ICB lysates were prepared and analyzed by immunoblotting using antibodies against IkB, p-IkB, and α-tubulin (used to normalize protein expression). (B) The optical density of the protein bands for IkB, p-IkB, and α-tubulin was analyzed. The results are presented as the mean ± SD of four rats in each group. The differences in the p-IkB/IkB ratios in ICB of rats from the groups were compared using ANOVA, followed by Tukey’s post hoc test. ns, not significant; *** *P* < 0.001 versus the control group; ^#^ *P* < 0.05; ^###^ *P* < 0.001.

The above results indicated that silibinin attenuated LPS-induced inflammatory reactions in rats with EIU. Furthermore, previous studies have shown that endotoxins or proinflammatory cytokines induce the phosphorylation and degradation of IκB, and then NF-κB, released from the inhibitory signalosome, translocates to the nucleus and induces the transcription of a number of genes, resulting in the expression of inflammatory proteins such as iNOS, COX-2, and ICAM-1.[19] Therefore, we tested whether NF-κB activation is suppressed by silibinin pretreatment in the ICBs of LPS-treated rats using western blotting. As shown in Fig 6, there was minimal expression of phosphorylated IkB in the ICBs of the control and Silibinin groups. Compared to the control group, the expression of phosphorylated IkB increased in the ICBs of the LPS group. By contrast, in the Silibinin (100)+ LPS group and Silibinin (200)+ LPS group, silibinin pretreatment reduced the increase of phosphorylated IkB expression in the ICBs of LPS-treated rats compared to the LPS group in a dose-responsive manner. Taken together, these results indicate that silibinin attenuates LPS-induced inflammatory reactions in rats with EIU, at least in part, by suppressing NF-κB activation.

### Silibinin attenuates LPS-induced inflammation by suppressing NF-kB activation in macrophages

**Fig 7:**
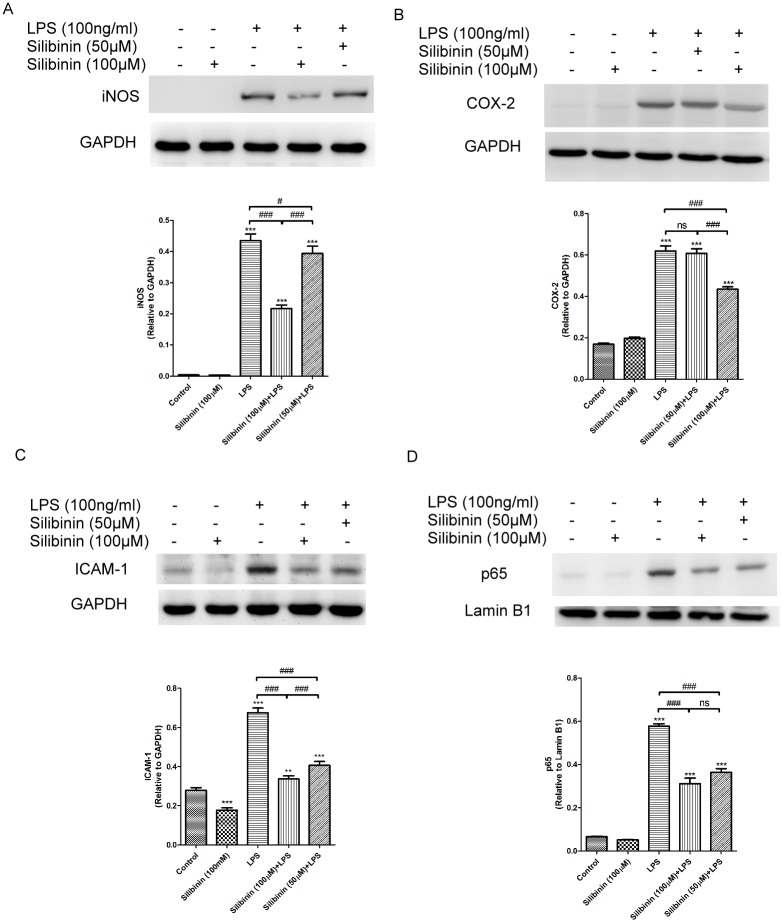
Silibinin inhibits Lipopolysaccharide (LPS)-induced Nitric Oxide Synthase (iNOS), Cyclooxygenase (COX-2), and Intercellular Adhesion Molecule (ICAM-1) expression in RAW cells by suppressing NF-kB activation. Cells were pretreated with 50 and 100 μM of silibinin for 18 h and then were cotreated with 100 ng/mL of LPS for 24 h. The cell lysates were collected to measure the expression of iNOS (A), COX-2 (B), and ICAM-1 (C) protein by western blotting. The optical density of the protein bands for iNOS, COX-2, ICAM-1, and glyceraldehyde 3-phosphate dehydrogenase (GAPDH) was analyzed. The results are presented as the mean ± SD of three independent experiments. The differences in the iNOS, COX-2, and ICAM-1 protein levels in RAW cells from the groups were compared using an ANOVA followed by Tukey’s post hoc test. ns, not significant; *** *P* < 0.001 versus the control group; ^#^ *P* < 0.05; ^###^ *P* < 0.001. (D) The nuclear translocation of p65 was determined in the nuclear extract of cell lysate by western blots using specific antibodies against p65. The optical density of the protein bands for p65 and lamin B1 was analyzed. The results are presented as the mean ± SD of three independent experiments. The differences in the p65 levels of RAW cells from the groups were compared using an ANOVA, followed by Tukey’s post hoc test. *** *P* < 0.001 versus the control group; ^##^ *P* < 0.05; ^###^ *P* < 0.001.

Previously, we found that macrophages participate in cellular infiltration into the anterior chamber and ICBs of LPS-treated rats.[14] Therefore, we used a mouse macrophage cell line (RAW 264.7 cells) as an *in vitro* model to explore the anti-inflammatory mechanism of silibinin on EIU. To investigate the anti-inflammatory effects of silibinin in RAW cells, we used western blotting to determine the expression of iNOS, COX-2, and ICAM-1 in LPS-treated cells. As shown in Fig 7A, 7B and 7C, compared to the control group, treatment with LPS significantly increased the expression of iNOS, COX-2, and ICAM-1 in RAW cells. However, silibinin (50 and 100 μM, respectively) decreased the expression of iNOS, COX-2, and ICAM-1 in LPS-treated RAW cells in a dose-responsive manner. These results suggest that silibinin attenuates LPS-induced inflammation in RAW cells. Considering that silibinin suppressed the activation of NF-kB in LPS-treated rats (Fig 6), NF-κB signaling could be involved in the silibinin-mediated inhibition of LPS-treated RAW cells. We collected the nuclear extract from LPS-treated RAW cells to examine the effect of silibinin on the expression of the nuclear p65 protein using western blot analysis. As seen in Fig 7D, treatment with LPS significantly increased the expression of nuclear p65 in RAW cells compared to the control group. By contrast, treatment with silibinin decreased the expression of nuclear p65 in LPS-treated RAW cells. These results suggest that silibinin suppresses LPS-induced NF-kB activation in RAW cells. Overall, these results demonstrate that silibinin attenuates LPS-induced inflammation, at least in part, by suppressing NF-kB activation in RAW cells.

## Discussion

Previously, we have reported that silibinin effectively suppresses TNF-α and IFN-γ-induced ICAM-1 expression and synthesis by inhibiting NF-κB activity in human RPE cells[23]; however, to the best of our knowledge, the effects of silibinin on EIU and its mechanism of action have not been clearly elucidated. In the present study, we have initiated an investigation into the anti-inflammatory effects and the possible mechanism employed by silibinin in a protective role against EIU *in vivo* and *in vitro*. The results of this study show that silibinin significantly attenuated the ocular inflammatory response in rats with EIU, with significant decreases in inflammatory cell infiltration, as well as protein, NO, and PG-E2 concentrations in the AqH. The expression of iNOS, COX-2, and ICAM-1 were also reduced by silibinin administration in the ICBs of LPS-treated rats. In addition, silibinin decreased the expression of phosphorylated IkB in the ICB of LPS-treated rats and consequently suppressed NF-kB activation. Furthermore, we also found that silibinin decreased the expression of iNOS, COX-2, ICAM-1, and nuclear p65 protein in LPS-treated RAW cells. Overall, these results suggest that silibinin attenuates the inflammatory responses of EIU in rats and RAW cells. Therefore, silibinin might be a potent preventative agent to treat acute ocular inflammation.

EIU is a model for acute inflammation, in which leukocytes, primarily neutrophils and monocytes, leave the iris venules and infiltrate the surrounding tissues.[15] Inflammatory mediators, including NO and PG-E2, participate in the pathogenesis of EIU.[27, 28] NO is synthesized by NO synthase (NOS) isoenzymes. In EIU, LPS induces iNOS expression in endothelial cells, macrophages, and polymorphonuclear leukocytes, which synthesize large amounts of NO and subsequently change hemodynamics and vascular permeability. [27, 29] Given that previous studies have demonstrated that suppressing the expression of iNOS can inhibit the development of EIU,[24, 29, 30] the activation of iNOS and the subsequent increased production of NO play important roles in the pathogenesis of EIU. In addition, after LPS is injected into rats, many tissues show increased COX-2 expression, [14, 31, 32] which participates in the production of proinflammatory prostaglandins.[33, 34] PG-E2, a major metabolite of COX-2, is an inflammatory mediator that contributes to the breakdown of the blood-aqueous barrier during EIU.[28] Given that the iNOS/NO and the COX-2/PG-E2 synthesis have additive effects in EIU, the inhibition of NO and PG-E2 synthesis has therapeutic effects on uveitis.[17] Therefore, to elucidate the mechanism responsible for the anti-inflammatory effect of silibinin pretreatment, we evaluated the effects of silibinin on the cell infiltration, protein concentration, and the levels of inflammatory mediators such as NO and PG-E2 in the AqH, and the enzymes, iNOS and COX-2, in the ICBs of rats with EIU. Consistent with previous studies,[17, 24, 28–30] our results showed that silibinin reduced the increase in cell infiltration, protein concentration, NO and PG-E2 production, and iNOS and COX-2 expression in rats with EIU. Taken together, these results also indicate that silibinin attenuates the increase in protein concentration in EIU of rats, possibly because silibinin influences both the induction of iNOS/NO and synthesis of COX-2/PG-E2. Consequently, these results may suggest that silibinin prevents LPS-induced breakdown of the blood-aqueous barrier integrity by decreasing the production of NO and PG-E2.

Previous studies have reported that the expression of ICAM-1 on the ciliary body is significantly augmented after LPS injection,[35] whereas interactions between ICAM-1 and lymphocyte functional antigen (LFA)-1 are mainly responsible for the adhesion of leukocytes before extravasation during EIU.[18, 36] Several studies have found that intraperitoneal injection of monoclonal antibodies against ICAM-1 inhibits LPS-induced cellular infiltration into the anterior segment.[18, 37] In the RPE cells in posterior uveitis patients, increased ICAM-1 expression has been detected, which facilitates the extravasation of inflammatory cells into the retina.[38] In EIU, the increase in TNF-α and IFN-γ expression play critical roles in this pathogenesis,[39–41] and in our previous study, we showed that silibinin inhibited TNF-α and IFN-γ-induced ICAM-1 gene expression and protein synthesis in RPE cells and suppressed TNF-α and IFN-γ-induced monocyte adhesion to RPE cells.[23] In the present study, we found that silibinin decreased LPS-induced cell infiltration in the anterior segment and ICAM-1 expression in the ICBs. Based on the aforementioned studies, these findings suggest that silibinin reduces infiltration of inflammatory cells, at least in part, by decreasing the ICAM-1 expression and subsequently affecting the interaction between ICAM-1 and LFA-1 *in vivo*. In addition, the interaction between ICAM-1 and LFA-1 can provide a second signal for T-cell activation, which plays an important role for T-cell migration to target tissues,[42] and T cells are essential for EIU.[10] Therefore, the data from this study suggest that silibinin attenuates LPS-induced immune cell activation by decreasing the ICAM-1 expression.

NF-κB is a heterodimer consisting of p50, p65, and the inhibitory subunit IκB, and resides in the cytoplasm. When cells are stimulated by endotoxins or proinflammatory cytokines, the IκB protein is phosphorylated and subsequently degraded.[43] In the absence of the inhibitory subunit, p50 and p65 are released and enter the nucleus to bind to the κB sequence to induce the transcription of a number of genes that promote the expression of inflammation-associated proteins such as ICAM-1, iNOS, and COX-2.[19] Silibinin is a potent inhibitor of the NF-κB signaling pathway that is responsible, in part, for the molecular basis for its anticancer, antiapoptotic, and anti-inflammatory effects.[44] In our previous studies, we showed that silibinin inhibited TNF-α and IFN-γ-induced ICAM-1 expression in human RPE cells by suppressing IκB phosphorylation and subsequently reducing translocation of the p65 subunit of NF-κB into the nucleus.[23] In this study, we report that silibinin suppresses LPS-induced phosphorylation of the IκB subunit of NF-κB, thereby preventing further NF-κB translocation *in vivo*. In addition, because the activation of NF-kB is critical for the induction of iNOS and COX-2 by LPS,[45, 46] our results note that silibinin decreases the expression of iNOS and COX-2 protein, and subsequently NO and PG-E2 production, by suppressing NF-κB activation. Given that the NF-kB signaling pathway plays an important role in the induction of ICAM-1 by LPS,[14] our results also show that silibinin reduced ICAM-1 expression by suppressing the activation of NF-kB. Overall, these results suggest that silibinin exerts its anti-inflammatory effect, at least in part, by suppressing phosphorylation of the IκB subunit of NF-κB.

In our previous study, we demonstrated that a majority of the inflammatory cells and macrophages infiltrated the ICBs and anterior chamber;[14] macrophages are associated with innate immunity and play a crucial role in the pathogenesis of EIU.[47, 48] After the systemic injection of LPS in the rat model of EIU, a large number of macrophages infiltrate the eyes, [49] which express iNOS and produce substantial amounts of NO in the retina and vitreous.[16] Therefore, we investigated the effects of silibinin on the dynamics of inflammatory mediators after stimulating RAW cells with LPS. To investigate the mechanism responsible for the anti-inflammatory effect of silibinin *in vitro*, we evaluated the effects of silibinin on the LPS-induced expression of proinflammatory mediators such as iNOS, COX-2, and ICAM-1 in RAW cells. Consistent with our *in vivo* results, silibinin attenuates the expression of proinflammatory mediators in LPS-treated RAW cells *in vitro*. Given that the expression of iNOS, COX-2, and ICAM-1 is regulated by NF-κB,[14, 45, 46] our previous studies also demonstrated that for a large number of nuclei positive staining of p65 were detected in LPS-treated RAW cells, indicative of NF-κB activation by LPS stimulation.[14] Therefore, we next investigated the role of NF-κB in the silibinin-mediated suppression of LPS-related inflammation in RAW cells *in vitro*. We used western blot analysis for p65 after nuclear extraction as a more accurate technique to quantify the levels of NF-κB activation in RAW cells. Consistent with our previous study, we found that silibinin decreased the expression of the nuclear p65 protein in LPS-treated RAW cells, suggesting that the LPS-induced NF-κB activation was suppressed by silibinin treatment. Taken together, these *in vitro* results indicate that the anti-inflammatory effects of silibinin were sequential events, at least in part, whereby the suppression of NF-kB activation led to a reduction in the LPS–induced expression of proinflammatory molecules.

LPS, a component of the outer membrane of Gram-negative bacteria, is one of the endotoxins that can cause death in rats when administered at a dosage of ~20 mg/kg.[8, 50] In the rat model of EIU, systemic injection of LPS at a dosage of ~200 μg/kg to 1mg/ kg is sufficient to produce ocular inflammation, but does not induce significant systemic diseases such as cardiac, hepatic, and renal diseases.[8, 51] Qin et al. report that footpad injection of LPS at 1 mg/kg induces moderate inflammation in the eyes without generating obvious hepatic and renal lesions.[51] Consequently, in this study, we injected LPS at a dosage of 300 μg/kg into the footpad to observe the effect of silibinin pretreatment on ocular inflammation. Consistent with previous studies, [8, 51] there were no significant systemic diseases after LPS injection at this dosage in the present study.

The limitations of the present study are that our results suggest the potential of silibinin in attenuating the cellular and molecular aspects of the inflammatory response in EIU of rats; however, treatment with silibinin prior to the induction of EIU, as performed in this study, is insufficient to suggest that silibinin suppresses ocular inflammation once it is established, because there is inadequate evidence to suggest that silibinin can reduce already established inflammation. As such, additional experiments, such as treatment with silibinin at the same time or after the induction of EIU, are needed to determine the effects of silibinin on established ocular inflammation.

In conclusion, we demonstrate that silibinin pretreatment on EIU *in vivo* and *in vitro* effectively attenuated the increase in cell infiltration and protein levels, the production of NO and PG-E2, and the expression of iNOS, COX-2, and ICAM-1, at least in part, by suppressing NF-kB activity. Furthermore, our study indicates that silibinin may be an ideal candidate for the development of a potential preventive agent to suppress ocular inflammatory diseases such as uveitis.

## References

[1] Durrani OM, Tehrani NN, Marr JE, Moradi P, Stavrou P, Murray PI. Degree, duration, and causes of visual loss in uveitis. The British journal of ophthalmology. 2004;88(9):1159–62. Epub 2004/08/20. 10.1136/bjo.2003.037226 15317708 PMC1772296

[2] Gritz DC, Wong IG. Incidence and prevalence of uveitis in Northern California; the Northern California Epidemiology of Uveitis Study. Ophthalmology. 2004;111(3):491–500; discussion Epub 2004/03/17. 10.1016/j.ophtha.2003.06.014 15019324

[3] Tomkins-Netzer O, Talat L, Bar A, Lula A, Taylor SR, Joshi L, et al Long-term clinical outcome and causes of vision loss in patients with uveitis. Ophthalmology. 2014;121(12):2387–92. Epub 2014/09/03. 10.1016/j.ophtha.2014.07.007 25178807

[4] Siddique SS, Shah R, Suelves AM, Foster CS. Road to remission: a comprehensive review of therapy in uveitis. Expert opinion on investigational drugs. 2011;20(11):1497–515. Epub 2011/09/23. 10.1517/13543784.2011.617741 21936708

[5] Pavesio CE, Decory HH. Treatment of ocular inflammatory conditions with loteprednol etabonate. The British journal of ophthalmology. 2008;92(4):455–9. Epub 2008/02/05. 10.1136/bjo.2007.132621 18245274

[6] Becker B, Mills DW. ELEVATED INTRAOCULAR PRESSURE FOLLOWING CORTICOSTEROID EYE DROPS. Jama. 1963;185:884–6. Epub 1963/09/14. 14043096 10.1001/jama.1963.03060110088027

[7] Jabs DA, Rosenbaum JT, Foster CS, Holland GN, Jaffe GJ, Louie JS, et al Guidelines for the use of immunosuppressive drugs in patients with ocular inflammatory disorders: recommendations of an expert panel. American journal of ophthalmology. 2000;130(4):492–513. Epub 2000/10/12. 11024423 10.1016/s0002-9394(00)00659-0

[8] Rosenbaum JT, McDevitt HO, Guss RB, Egbert PR. Endotoxin-induced uveitis in rats as a model for human disease. Nature. 1980;286(5773):611–3. Epub 1980/08/07. 7402339 10.1038/286611a0

[9] Howes EL Jr., Aronson SB, McKay DG. Ocular vascular permeability. Effect of systemic administration of bacterial endotoxin. Archives of ophthalmology (Chicago, Ill: 1960). 1970;84(3):360–7. Epub 1970/09/01. 10.1001/archopht.1970.00990040362017 4917598

[10] Kogiso M, Tanouchi Y, Mimura Y, Nagasawa H, Himeno K. Endotoxin-induced uveitis in mice. 1. Induction of uveitis and role of T lymphocytes. Japanese journal of ophthalmology. 1992;36(3):281–90. Epub 1992/01/01. 1361208

[11] Okumura A, Mochizuki M, Nishi M, Herbort CP. Endotoxin-induced uveitis (EIU) in the rat: a study of inflammatory and immunological mechanisms. International ophthalmology. 1990;14(1):31–6. Epub 1990/01/01. 1691157 10.1007/BF00131166

[12] da Silva PS, Girol AP, Oliani SM. Mast cells modulate the inflammatory process in endotoxin-induced uveitis. Molecular vision. 2011;17:1310–9. Epub 2011/06/03. 21633711 PMC3103740

[13] Yadav UC, Ramana KV. Endotoxin-induced uveitis in rodents. Methods in molecular biology (Clifton, NJ). 2013;1031:155–62. Epub 2013/07/05. 10.1007/978-1-62703-481-4_18 23824898

[14] Chang YH, Horng CT, Chen YH, Chen PL, Chen CL, Liang CM, et al Inhibitory effects of glucosamine on endotoxin-induced uveitis in Lewis rats. Investigative ophthalmology & visual science. 2008;49(12):5441–9. Epub 2008/08/23. 18719082 10.1167/iovs.08-1784

[15] Bhattacherjee P, Williams RN, Eakins KE. An evaluation of ocular inflammation following the injection of bacterial endotoxin into the rat foot pad. Investigative ophthalmology & visual science. 1983;24(2):196–202. Epub 1983/02/01. 6337969

[16] Jacquemin E, de Kozak Y, Thillaye B, Courtois Y, Goureau O. Expression of inducible nitric oxide synthase in the eye from endotoxin-induced uveitis rats. Investigative ophthalmology & visual science. 1996;37(6):1187–96. Epub 1996/05/01. 8631633

[17] Bellot JL, Palmero M, Garcia-Cabanes C, Espi R, Hariton C, Orts A. Additive effect of nitric oxide and prostaglandin-E2 synthesis inhibitors in endotoxin-induced uveitis in the rabbit. Inflammation research: official journal of the European Histamine Research Society [et al]. 1996;45(4):203–8. Epub 1996/04/01. 10.1007/BF02285162 8741011

[18] Becker MD, Garman K, Whitcup SM, Planck SR, Rosenbaum JT. Inhibition of leukocyte sticking and infiltration, but not rolling, by antibodies to ICAM-1 and LFA-1 in murine endotoxin-induced uveitis. Investigative ophthalmology & visual science. 2001;42(11):2563–6. Epub 2001/10/03. 11581199

[19] Sahnoun Z, Jamoussi K, Zeghal KM. [Free radicals and antioxidants: physiology, human pathology and therapeutic aspects (part II)]. Therapie. 1998;53(4):315–39. Epub 1998/11/07. 9806002

[20] Baeuerle PA, Henkel T. Function and activation of NF-kappa B in the immune system. Annual review of immunology. 1994;12:141–79. Epub 1994/01/01. 10.1146/annurev.iy.12.040194.001041 8011280

[21] Surai PF. Silymarin as a Natural Antioxidant: An Overview of the Current Evidence and Perspectives. Antioxidants (Basel, Switzerland). 2015;4(1):204–47. Epub 2016/01/20. 10.3390/antiox4010204 PMC4665566 26785346

[22] Ramasamy K, Agarwal R. Multitargeted therapy of cancer by silymarin. Cancer letters. 2008;269(2):352–62. Epub 2008/05/13. 10.1016/j.canlet.2008.03.053 18472213 PMC2612997

[23] Chen YH, Chen CL, Liang CM, Liang JB, Tai MC, Chang YH, et al Silibinin inhibits ICAM-1 expression via regulation of N-linked and O-linked glycosylation in ARPE-19 cells. BioMed research international. 2014;2014:701395 Epub 2014/07/18. 10.1155/2014/701395 25032222 PMC4083610

[24] Goureau O, Bellot J, Thillaye B, Courtois Y, de Kozak Y. Increased nitric oxide production in endotoxin-induced uveitis. Reduction of uveitis by an inhibitor of nitric oxide synthase. Journal of immunology (Baltimore, Md: 1950). 1995;154(12):6518–23. Epub 1995/06/15. 7539024

[25] Mitchell JA, Warner TD. Cyclo-oxygenase-2: pharmacology, physiology, biochemistry and relevance to NSAID therapy. British journal of pharmacology. 1999;128(6):1121–32. Epub 1999/12/01. 10.1038/sj.bjp.0702897 10578123 PMC1571744

[26] Chen JT, Liang JB, Chou CL, Chien MW, Shyu RC, Chou PI, et al Glucosamine sulfate inhibits TNF-alpha and IFN-gamma-induced production of ICAM-1 in human retinal pigment epithelial cells in vitro. Investigative ophthalmology & visual science. 2006;47(2):664–72. Epub 2006/01/25. 16431966 10.1167/iovs.05-1008

[27] Tilton RG, Chang K, Corbett JA, Misko TP, Currie MG, Bora NS, et al Endotoxin-induced uveitis in the rat is attenuated by inhibition of nitric oxide production. Investigative ophthalmology & visual science. 1994;35(8):3278–88. Epub 1994/07/01. 7519183

[28] Smith JR, Hart PH, Williams KA. Basic pathogenic mechanisms operating in experimental models of acute anterior uveitis. Immunology and cell biology. 1998;76(6):497–512. Epub 1999/01/20. 10.1046/j.1440-1711.1998.00783.x 9893027

[29] Kamata K, Inazu M, Takeda H, Goto H, Matsumiya T, Usui M. Effect of a selective inducible nitric oxide synthase inhibitor on intraocular nitric oxide production in endotoxin-induced uveitis rabbits: in vivo intraocular microdialysis study. Pharmacological research. 2003;47(6):485–91. Epub 2003/05/14. 12742001 10.1016/s1043-6618(03)00057-4

[30] Mandai M, Yoshimura N, Yoshida M, Iwaki M, Honda Y. The role of nitric oxide synthase in endotoxin-induced uveitis: effects of NG-nitro L-arginine. Investigative ophthalmology & visual science. 1994;35(10):3673–80. Epub 1994/09/01. 7522226

[31] Cao C, Matsumura K, Yamagata K, Watanabe Y. Induction by lipopolysaccharide of cyclooxygenase-2 mRNA in rat brain; its possible role in the febrile response. Brain research. 1995;697(1–2):187–96. Epub 1995/10/30. 8593576 10.1016/0006-8993(95)00839-i

[32] Ichitani Y, Holmberg K, Maunsbach AB, Haeggstrom JZ, Samuelsson B, De Witt D, et al Cyclooxygenase-1 and cyclooxygenase-2 expression in rat kidney and adrenal gland after stimulation with systemic lipopolysaccharide: in situ hybridization and immunocytochemical studies. Cell and tissue research. 2001;303(2):235–52. Epub 2001/04/09. 11291770 10.1007/s004410000296

[33] Masferrer JL, Zweifel BS, Manning PT, Hauser SD, Leahy KM, Smith WG, et al Selective inhibition of inducible cyclooxygenase 2 in vivo is antiinflammatory and nonulcerogenic. Proceedings of the National Academy of Sciences of the United States of America. 1994;91(8):3228–32. Epub 1994/04/12. 8159730 10.1073/pnas.91.8.3228 PMC43549

[34] Seibert K, Zhang Y, Leahy K, Hauser S, Masferrer J, Perkins W, et al Pharmacological and biochemical demonstration of the role of cyclooxygenase 2 in inflammation and pain. Proceedings of the National Academy of Sciences of the United States of America. 1994;91(25):12013–7. Epub 1994/12/06. 7991575 10.1073/pnas.91.25.12013 PMC45366

[35] Kanagawa T, Matsuda S, Mikawa Y, Kogiso M, Nagasawa H, Himeno K, et al Role of ICAM-1 and LFA-1 in endotoxin-induced uveitis in mice. Japanese journal of ophthalmology. 1996;40(2):174–80. Epub 1996/01/01. 8876384

[36] Springer TA. Traffic signals for lymphocyte recirculation and leukocyte emigration: the multistep paradigm. Cell. 1994;76(2):301–14. Epub 1994/01/28. 7507411 10.1016/0092-8674(94)90337-9

[37] Whitcup SM, Hikita N, Shirao M, Miyasaka M, Tamatani T, Mochizuki M, et al Monoclonal antibodies against CD54 (ICAM-1) and CD11a (LFA-1) prevent and inhibit endotoxin-induced uveitis. Experimental eye research. 1995;60(6):597–601. Epub 1995/06/01. 7641842 10.1016/s0014-4835(05)80001-6

[38] Whitcup SM, Chan CC, Li Q, Nussenblatt RB. Expression of cell adhesion molecules in posterior uveitis. Archives of ophthalmology (Chicago, Ill: 1960). 1992;110(5):662–6. Epub 1992/05/01. 10.1001/archopht.1992.01080170084029 1374609

[39] de Vos AF, Klaren VN, Kijlstra A. Expression of multiple cytokines and IL-1RA in the uvea and retina during endotoxin-induced uveitis in the rat. Investigative ophthalmology & visual science. 1994;35(11):3873–83. Epub 1994/10/01. 7928184

[40] Yoshida M, Yoshimura N, Hangai M, Tanihara H, Honda Y. Interleukin-1 alpha, interleukin-1 beta, and tumor necrosis factor gene expression in endotoxin-induced uveitis. Investigative ophthalmology & visual science. 1994;35(3):1107–13. Epub 1994/03/01. 8125721

[41] de Vos AF, van Haren MA, Verhagen C, Hoekzema R, Kijlstra A. Kinetics of intraocular tumor necrosis factor and interleukin-6 in endotoxin-induced uveitis in the rat. Investigative ophthalmology & visual science. 1994;35(3):1100–6. Epub 1994/03/01. 8125720

[42] Grakoui A, Bromley SK, Sumen C, Davis MM, Shaw AS, Allen PM, et al The immunological synapse: a molecular machine controlling T cell activation. Science (New York, NY). 1999;285(5425):221–7. Epub 1999/07/10. 10.1126/science.285.5425.221 10398592

[43] Baldwin AS Jr. The NF-kappa B and I kappa B proteins: new discoveries and insights. Annual review of immunology. 1996;14:649–83. Epub 1996/01/01. 10.1146/annurev.immunol.14.1.649 8717528

[44] Gazak R, Walterova D, Kren V. Silybin and silymarin—new and emerging applications in medicine. Current medicinal chemistry. 2007;14(3):315–38. Epub 2007/02/20. 17305535 10.2174/092986707779941159

[45] Xie QW, Kashiwabara Y, Nathan C. Role of transcription factor NF-kappa B/Rel in induction of nitric oxide synthase. The Journal of biological chemistry. 1994;269(7):4705–8. Epub 1994/02/18. 7508926

[46] Noh EJ, Ahn KS, Shin EM, Jung SH, Kim YS. Inhibition of lipopolysaccharide-induced iNOS and COX-2 expression by dehydroevodiamine through suppression of NF-kappaB activation in RAW 264.7 macrophages. Life sciences. 2006;79(7):695–701. Epub 2006/03/24. 10.1016/j.lfs.2006.02.020 16554073

[47] Marie O, Thillaye-Goldenberg B, Naud MC, de Kozak Y. Inhibition of endotoxin-induced uveitis and potentiation of local TNF-alpha and interleukin-6 mRNA expression by interleukin-13. Investigative ophthalmology & visual science. 1999;40(10):2275–82. Epub 1999/09/07. 10476793

[48] Lemaitre C, Thillaye-Goldenberg B, Naud MC, de Kozak Y. The effects of intraocular injection of interleukin-13 on endotoxin-induced uveitis in rats. Investigative ophthalmology & visual science. 2001;42(9):2022–30. Epub 2001/08/02. 11481267

[49] Yang P, de Vos AF, Kijlstra A. Macrophages in the retina of normal Lewis rats and their dynamics after injection of lipopolysaccharide. Investigative ophthalmology & visual science. 1996;37(1):77–85. Epub 1996/01/01. 8550337

[50] Thomas L. The physiological disturbances produced by endotoxins. Annual review of physiology. 1954;16:467–90. Epub 1954/01/01. 10.1146/annurev.ph.16.030154.002343 13171836

[51] Qin YJ, Chu KO, Yip YW, Li WY, Yang YP, Chan KP, et al Green tea extract treatment alleviates ocular inflammation in a rat model of endotoxin-induced uveitis. PloS one. 2014;9(8):e103995 Epub 2014/08/06. 10.1371/journal.pone.0103995 25093862 PMC4122397

